# *PITX2* Modulates Atrial Membrane Potential and the Antiarrhythmic Effects of Sodium-Channel Blockers

**DOI:** 10.1016/j.jacc.2016.07.766

**Published:** 2016-10-25

**Authors:** Fahima Syeda, Andrew P. Holmes, Ting Y. Yu, Samantha Tull, Stefan Michael Kuhlmann, Davor Pavlovic, Daniel Betney, Genna Riley, Jan P. Kucera, Florian Jousset, Joris R. de Groot, Stephan Rohr, Nigel A. Brown, Larissa Fabritz, Paulus Kirchhof

**Affiliations:** a Institute of Cardiovascular Sciences, University of Birmingham, Birmingham, United Kingdom; b Physical Sciences of Imaging in the Biomedical Sciences, School of Chemistry, University of Birmingham, Birmingham, United Kingdom; c Department of Physiology, University of Bern, Bern, Switzerland; d Heart Center, Department of Cardiology, Academisch Medisch Centrum, Amsterdam, the Netherlands; e St. George’s Hospital Medical School, University of London, London, United Kingdom; f Department of Cardiovascular Medicine, University Hospital Muenster, Muenster, Germany; g Atrial Fibrillation NETwork, Muenster, Germany; h University Hospitals Birmingham NHS Foundation Trust, Birmingham, United Kingdom; i Sandwell and West Birmingham Hospitals NHS Trust, Birmingham, United Kingdom

**Keywords:** antiarrhythmic drugs, atrial fibrillation, drug targets, electrophysiology, personalized medicine, rhythm control, AAD, antiarrhythmic drug, APD, action potential duration, ERP, effective refractory period, HEK, human embryonic kidney, LA, left atrium, LAA, left atrial appendage, mRNA, messenger ribonucleic acid, Na, sodium, *PITX2*, paired like homeodomain-2, PRR, post-repolarization refractoriness, RMP, resting membrane potential, SNP, single nucleotide polymorphism, TASK-2, TWIK-related acid-sensitive K^+^ channel

## Abstract

**Background:**

Antiarrhythmic drugs are widely used to treat patients with atrial fibrillation (AF), but the mechanisms conveying their variable effectiveness are not known. Recent data suggested that paired like homeodomain-2 transcription factor (*PITX2*) might play an important role in regulating gene expression and electrical function of the adult left atrium (LA).

**Objectives:**

After determining LA *PITX2* expression in AF patients requiring rhythm control therapy, the authors assessed the effects of *Pitx2c* on LA electrophysiology and the effect of antiarrhythmic drugs.

**Methods:**

LA *PITX2* messenger ribonucleic acid (mRNA) levels were measured in 95 patients undergoing thoracoscopic AF ablation. The effects of flecainide, a sodium (Na^+^)-channel blocker, and d,l-sotalol, a potassium channel blocker, were studied in littermate mice with normal and reduced *Pitx2c* mRNA by electrophysiological study, optical mapping, and patch clamp studies. *PITX2*-dependent mechanisms of antiarrhythmic drug action were studied in human embryonic kidney (HEK) cells expressing human Na channels and by modeling human action potentials.

**Results:**

Flecainide 1 μmol/l was more effective in suppressing atrial arrhythmias in atria with reduced *Pitx2c* mRNA levels (*Pitx2c*^+/–^). Resting membrane potential was more depolarized in *Pitx2c*^+/–^ atria, and TWIK-related acid-sensitive K^+^ channel 2 (TASK-2) gene and protein expression were decreased. This resulted in enhanced post-repolarization refractoriness and more effective Na-channel inhibition. Defined holding potentials eliminated differences in flecainide’s effects between wild-type and *Pitx2c*^+/–^ atrial cardiomyocytes. More positive holding potentials replicated the increased effectiveness of flecainide in blocking human Na_v_1.5 channels in HEK293 cells. Computer modeling reproduced an enhanced effectiveness of Na-channel block when resting membrane potential was slightly depolarized.

**Conclusions:**

*PITX2* mRNA modulates atrial resting membrane potential and thereby alters the effectiveness of Na-channel blockers. *PITX2* and ion channels regulating the resting membrane potential may provide novel targets for antiarrhythmic drug development and companion therapeutics in AF.

## Central Illustration

**Figure:**
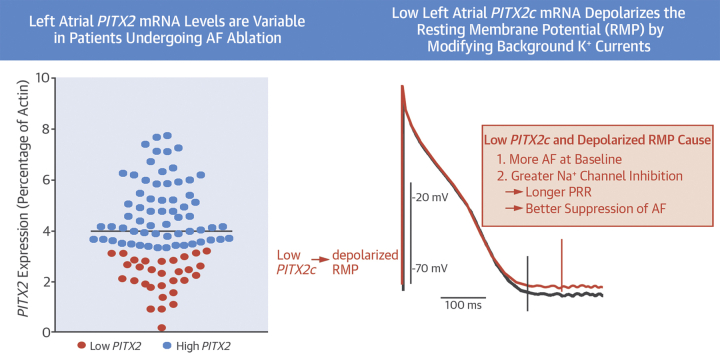

Atrial fibrillation (AF) causes cardiovascular death, frequent hospitalization, and cognitive decline even in patients treated according to guidelines 1, 2, 3. Antiarrhythmic drug (AAD) therapy remains the most commonly used treatment to maintain sinus rhythm in AF patients, but AAD effectiveness remains limited (3). Unfortunately, we lack a basic understanding of why AADs prevent AF over long periods in some patients but not in others 4, 5. Identifying factors that modify the effects of AADs would allow the selection of responsive patients and could help guide development of novel AADs (6).

Paired like homeodomain-2 transcription factor (*PITX2*) is a transcription factor that regulates the development of the left atrium (LA) and thoracic organs. Its c isoform is expressed in the adult LA and regulates the expression of LA ion channels 7, 8, 9. Low atrial *Pitx2* expression renders mice susceptible to AF and shortens the LA action potential 8, 10, 11. In this study, we investigated how atrial *PITX2* modifies the effects of AADs.

We detected variable LA *PITX2* messenger ribonucleic acid (mRNA) expression in AF patients requiring rhythm control therapy. After finding that low *Pitx2c* enhanced the effect of flecainide, mediated by a more positive resting membrane potential (RMP), we identified reduced TWIK-related acid-sensitive K^+^ channel 2 (TASK-2) expression as a possible driver of this effect and replicated these effects in cells expressing human sodium (Na) channels and in a human atrial action potential model.

## Methods

All experiments were conducted under the Animals (Scientific Procedures) Act 1986, and approved by the home office (PPL number 30/2967) and the institutional review board at the University of Birmingham. Analyses of human atrial tissue were approved by the institutional review board of Academic Medical Center, Amsterdam, the Netherlands. All patients provided written informed consent.

Left atrial appendages (LAAs) were excised from 95 patients undergoing bilateral thoracoscopic AF ablation either in the AFACT (Atrial Fibrillation Ablation and Autonomic Modulation via Thoracoscopic Surgery) trial (12) or undergoing similar procedures in the same centers using an endoscopic stapling device, snap frozen in liquid nitrogen and stored at –80°C (13). Deoxyribonucleic acid and ribonucleic acid were extracted using DNeasy and RNeasy kits (Qiagen Ltd., Manchester, United Kingdom), respectively. *PITX2* mRNA content was quantified by quantitative polymerase chain reaction. Single nucleotide polymorphisms (SNPs) rs2200733, rs6838973, and rs1448818 (14) were identified using TaqMan assays (Thermo Fisher Scientific Inc., Waltham, Massachusetts).

Adult mice (age 12 to 16 weeks) on an MF1 background with normal or reduced (*Pitx2c*^+/−^) atrial *Pitx2c* expression were studied (8).

LA epicardial monophasic action potentials were recorded from Langendorff-perfused murine hearts 8, 15. Programmed stimulation was performed at baseline and with flecainide 1 μmol/l or d,l-sotalol 10 μmol/l. Arrhythmia inducibility and effective refractory period (ERP) were measured by using single right atrial extrastimuli after steady-state pacing in 1-ms decrements 15, 16, 17, 18. Transmembrane action potentials were recorded using borosilicate glass microelectrodes from superfused murine LAs (17), RMP, action potential duration (APD), upstroke velocity, and activation times were analyzed 15, 17, 18.

The human atrial cell model of Courtemanche et al. (19) was used. *Pitx2c*^+/–^ deficiency was modeled by reducing I_K1_ conductance by 25% and doubling I_Kr_ conductance. Simulations were run in strands of 100 atrial cells (cell length 100 μm). The 5 leftmost cells of the strand were paced (S1) for 2 min at 1,000- and 500-ms basic cycle lengths. Premature stimulation (S2) was applied to determine the ERP and conduction velocity as measured from cells 25 to 75. Values for all other parameters were measured from the 50th cell. For the modeling, post-repolarization refractoriness (PRR) was calculated as the difference between APD at –60 mV repolarization and ERP.

LA cell isolation was performed as previously reported (20). Standard I_Na_ and I_K1_ currents were recorded as previously published 18, 19, 20. Background K^+^ (TASK-like) currents sensitive to high Ba^2+^ (10 mM) were measured 21, 22, 23. Human embryonic kidney (HEK) 293 cells stably expressing the human Na_v_1.5 channel were obtained (SB Ion Channels, Glasgow, UK).

Ribonucleic acid and complementary deoxyribonucleic acid were synthesized from murine LA, (SuperScript VILO, Thermo Fisher Scientific Inc.) to quantify expression of 20 atrial ion channels and genes with suspected *PITX2*-dependent regulation (9) using custom-designed Taqman low density array plates (Thermo Fisher Scientific Inc.). Western immunoblotting was performed on murine LA tissue lysates with antibodies detecting TASK-2, K_v_1.6, Na/K ATPase alpha-1, Na/K ATPase alpha-2, Na/Ca exchanger 1, Serca2a, Na_v_1.5, or calnexin, using standard methods.

Optical action potentials and calcium ion (Ca^2+^) transients were recorded in murine LA and analyzed using custom-made MATLAB algorithms (MathWorks, Natick, Massachusetts) as previously described (17).

### Statistical analysis

All experiments were performed and analyzed in a blinded fashion. Murine studies were performed and analyzed blinded to genotype in littermate pairs. Categorical data were compared using the Fisher exact test. Numerical data were compared by 2-sided paired parametric Student *t* tests (e.g., measurements before and after perfusion of flecainide or sotalol) and Wilcoxon signed rank tests. Multiple measurements were assessed by repeated measures of analysis of variance followed by correction for multiple comparison (Bonferroni test) if the overall test was significant. Two-sided p < 0.05 were considered significant. Box plots depict individual measurements (points), mean, and SEM. Statistics and figures were created using Prism 5 (GraphPad Software, San Diego, California).

## Results

*PITX2* mRNA varied markedly in human LAA (Central Illustration) harvested from AF patients (Table 1) (13), suggesting that a 50% lowered *PITX2* expression defines a large, potentially clinically relevant group of AF patients. This did not directly correlate with SNP haplotype (Table 2), although we found numerically lower *PITX2c* levels in patients with 5 risk alleles.

**Figure 1:**
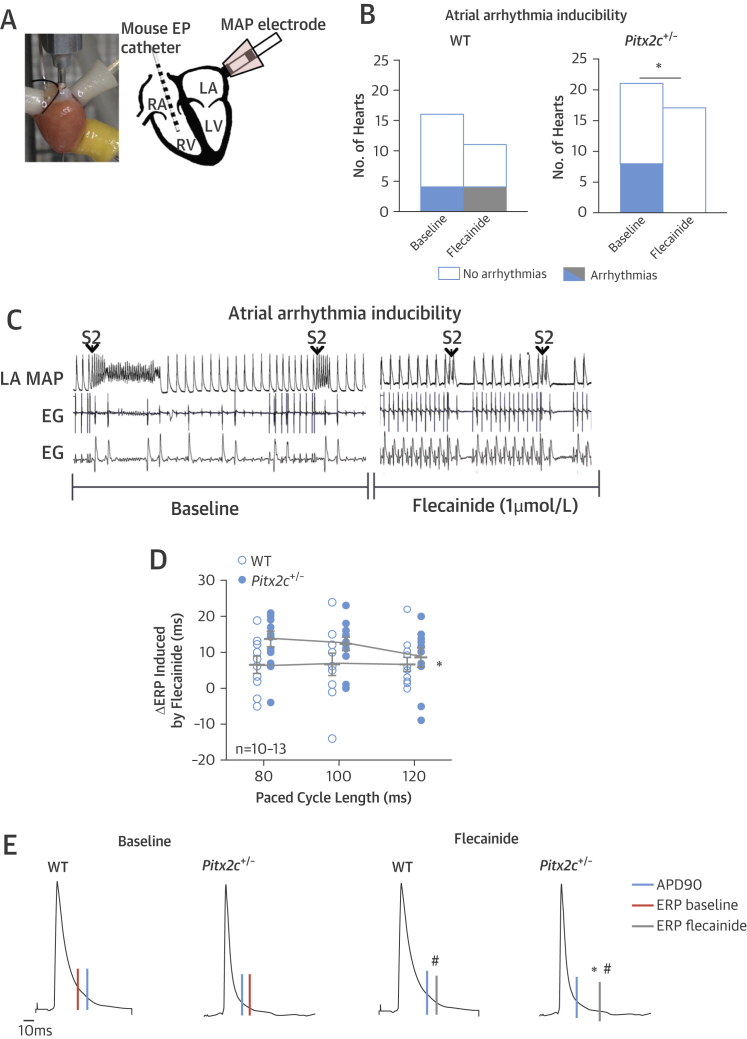
Atrial Arrhythmia Inducibility in *Pitx2c*^+/–^ Murine Whole Hearts **(A)** Image and schematic representation of the Langendorff-perfused heart. **(B)** Atrial arrhythmia inducibility in isolated, beating hearts from wild-type (WT) and reduced paired like homeodomain 2 messenger ribonucleic acid (*Pitx2c*^*+/*–^) mice. Flecainide abolished atrial arrhythmia inducibility in *Pitx2c*^+/–^ hearts only. *p < 0.05 flecainide versus baseline. **(C)** Representative trace of atrial fibrillation (AF) induced during programmed stimulation at baseline, showing reduced severity of arrhythmias with 1 μmol/l flecainide in *Pitx2c*^+/–^ atria. **(D)** Effects of flecainide on atrial effective refractory period (ERP) in wild-type and *Pitx2c*^+/–^ isolated, beating hearts. Shown is the difference in atrial ERP between baseline and 1 μmol/l flecainide at 80- to 120-ms paced cycle length following a single extrastimulus (S2) in WT and *Pitx2c*^+/–^ isolated, beating hearts. *p < 0.05 between genotypes across all cycle lengths. **(E)** Whereas flecainide prolonged ERP in both genotypes, this effect was more pronounced in *Pitx2c*^+/–^ atria. Flecainide caused post-repolarization refractoriness (PRR), the difference between ERP **(orange and grey lines)** and APD_90_**(blue lines)**, in WT and *Pitx2c*^+/–^ atria. Flecainide-induced PRR in *Pitx2c*^+/–^ is almost 3 times that of WT atria. *p < 0.05 WT versus *Pitx2c*^+/–^. ^#^p < 0.05 baseline versus 1 μmol/l flecainide. APD = action potential duration; EG = intracardiac electrogram; EP = electrophysiology; LA = left atrium; LV = left ventricle; MAP = monophasic action potential; RA = right atrium; RV = right ventricle.

Flecainide suppressed atrial arrhythmias in murine *Pitx2c*^+/–^ hearts. Flecainide abolished induced atrial arrhythmias in hearts with reduced *Pitx2c* expression (0 of 17 hearts with atrial arrhythmias) but not in hearts with normal *Pitx2c* expression (atrial arrhythmias remained in 3 of 12 hearts) (Figures 1A to 1C). Flecainide prolonged ERPs and refractoriness beyond the end of repolarization (PRR) calculated as the difference between ERP and APD_90_ (ms). Flecainide prolonged PRR more in hearts with reduced *Pitx2c* expression (Figures 1D and 1E, Table 3). *PITX2c*^+/–^ hearts had shorter atrial action potentials (8). Flecainide abolished APD differences between *Pitx2c*^+/–^ and wild-type LA by prolonging early repolarization (APD_30_, APD_50_, and APD_70_) (Table 3). Murine atrial *PITX2* expression did not modulate the effects of sotalol on atrial APD or ERP (Table 4).

**Figure 2:**
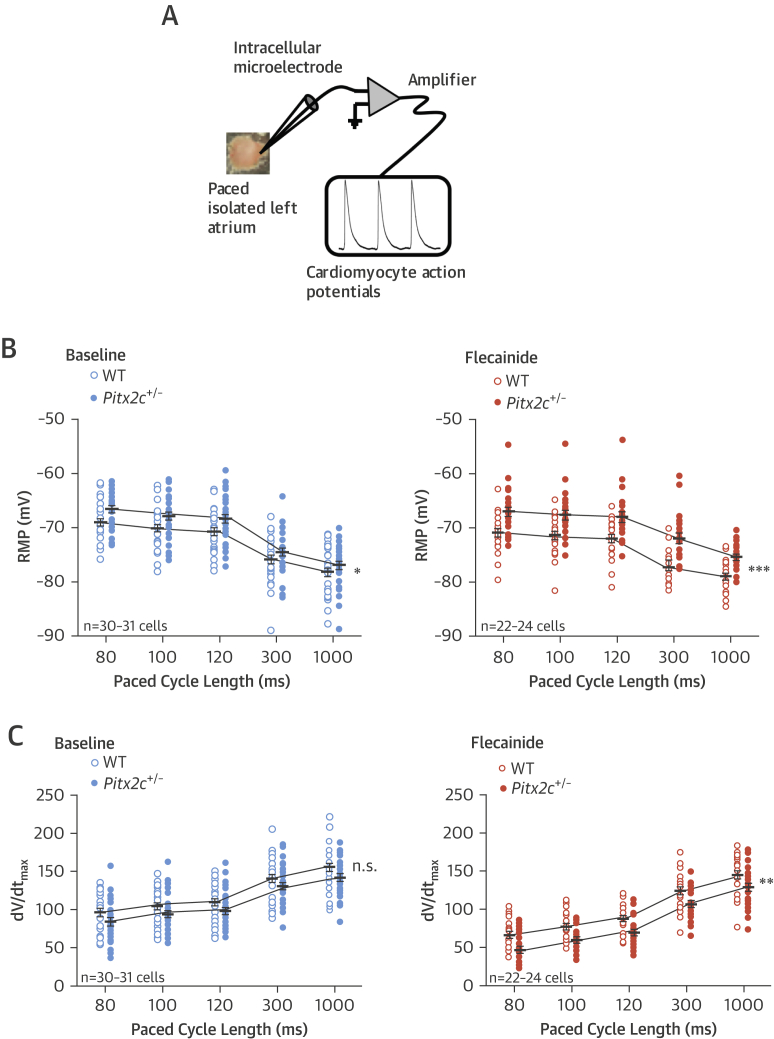
Resting Membrane Potential and Maximum Upstroke Velocity **(A)** Schematic representation of sharp microelectrode electrode recordings from the superfused whole LA. **(B)** Resting membrane potential (RMP) in WT and *Pitx2c*^+/–^ LA at baseline and with 1 μmol/l flecainide. *Pitx2c*^+/–^ LA have depolarized RMP. The difference between WT and *Pitx2c*^+/–^ is exaggerated with flecainide. *p < 0.05; ***p < 0.001 across all cycle lengths. **(C)** Flecainide unmasked a lower maximum upstroke velocity (dV/dt_max_) in *Pitx2c*^+/–^ LA compared to WT. **p < 0.01 versus WT. Abbreviations as in Figure 1.

RMP was slightly depolarized in LA murine cells with reduced *Pitx2c* expression (range of mean depolarization 1.2 to 2.4 mV over 5 cycle lengths; all p < 0.05) (Figures 2A and 2B). Atrial *Pitx2c* levels did not significantly affect dV/dt_max_ (100-ms paced cycle length: wild-type: 104.4 ± 4.3 V/s; *Pitx2c*^+/–^: 93.7 ± 4.5 V/s) (Figure 2C). Flecainide did not modify atrial RMP (Figure 2B) but reduced action potential amplitude consistent with its Na-channel blocking effect, specifically at 100-ms cycle length: wild-type baseline: 77.5 ± 1.2 mV (n = 30); wild-type flecainide: 71.3 ± 1.2 mV (n = 31); *Pitx2c*^+/–^ baseline: 73.4 ± 1.3 mV (n = 22); and *Pitx2c*^+/–^ flecainide: 65.1 ± 1.45 mV (n = 24).

**Figure 3:**
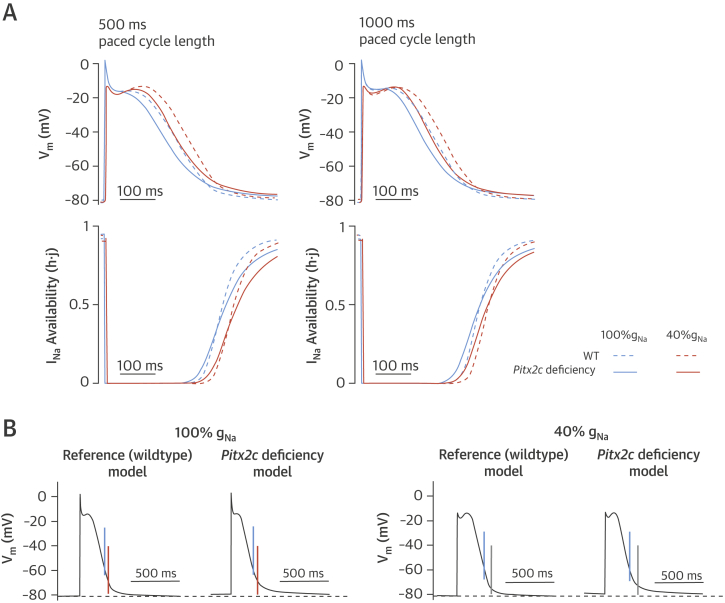
Modeling of the Electrophysiological Consequences of *Pitx2c* Deficiency **(A)** Propagated action potentials **(top)** simulated with the Courtemanche–Ramirez–Nattel model modified to reflect the more positive RMP of murine *Pitx2c*^+/–^ atria and the effect of 60% sodium current (I_Na_) block at pacing cycle lengths of 500 and 1,000 ms, with 2-min pre-pacing, and corresponding time courses of the product of the 2 inactivation gates *h* and *j* of I_Na_**(bottom),** reflecting I_Na_ availability. **(B)** Reduced sodium conductance (g_Na_) increased post-repolarization refractoriness (PRR) in the reference model and the *Pitx2c* deficiency model. After reducing g_Na_, PRR was greater in the *Pitx2c* deficiency model than in the reference model **(grey lines)**. Lines denote APD_60_**(blue)** and ERP (**orange**: with 100% gNa; **grey**: with 40% gNa). Abbreviations as in Figures 1 and 2.

Because the Courtemanche–Ramirez–Nattel model does not incorporate background K^+^ currents (19), we simulated a depolarized RMP in this model by a 25% reduction in I_K1_. This reduced the RMP at 500-ms paced cycle length by 2 mV from 79.9 mV (“normal *PITX2*”) to –77.9 mV (“low *PITX2*”). Na channels recovered from inactivation more slowly upon partial I_Na_ block (50% or 60%) (Figure 3A). Furthermore, PRR was enhanced in the *PITX2* deficiency model (Figure 3B and Table 5). Inhibition of I_Na_ reduced upstroke velocity (dV/dt_max_) and conduction velocity in both models, and reproduced the prolongation of PRR (Figure 3B).

**Figure 4:**
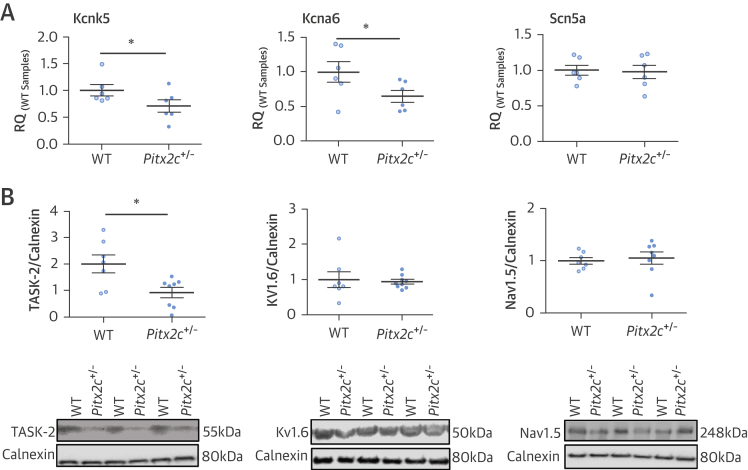
mRNA and Protein Expression in Murine LA **(A)** Relative quantity (RQ) of messenger ribonucleic acid (mRNA) of selected ion channels in LA from *Pitx2c*^+/–^ and WT mice. Expression levels were measured relative to WT sample 1. Kcnk5 encodes TWIK-related acid-sensitive K^+^ channel 2 (TASK-2), Kcna6 encodes K_v_1.6, and Scn5a encodes Na_v_1.5. *p < 0.05. **(B)** Concentration of TASK-2, K_V_1.6, and Na_v_1.5 proteins relative to calnexin (arbitrary units). Representative immunoblots are displayed below the corresponding dot plot. *p < 0.05. Abbreviations as in Figures 1 and 2.

*Kcna6* and *Kcnk5* mRNA expression were reduced in *Pitx2c*^+/–^ murine LA (Figure 4A, Online Table 1), whereas mRNA concentrations of 20 other ion channels or related genes were not altered. Kv1.6 protein concentration was unaltered, whereas TASK-2 protein concentration was reduced in murine atria with reduced *Pitx2c* expression (Figure 4B). Na_v_1.5 mRNA and protein expression were not changed (Figures 4A and 4B).

**Figure 5:**
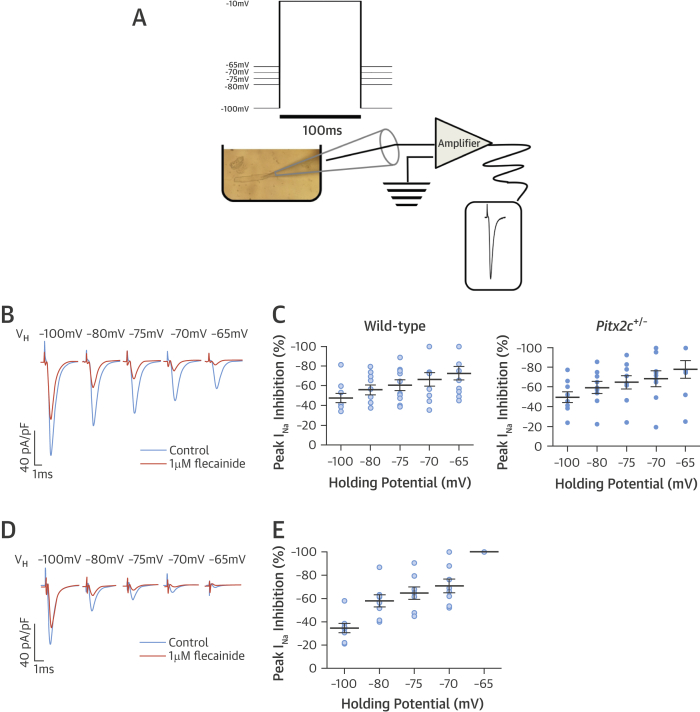
Effect of Membrane Potential on Sodium-Channel Inhibition **(A)** Schematic representation of patch clamp experiments carried out in isolated atrial cardiomyocytes. Human embryonic kidney (HEK) cells were patch clamped using the same setup. **(B)** Representative traces showing I_Na_ measured by patch clamping in isolated atrial cardiomyocytes, at baseline and with flecainide, at increasing holding potentials (–100 to –65 mV). **(C)** Reduction in peak I_Na_ was enhanced with increased holding potentials, irrespective of cardiomyocyte origin. **(D)** Representative traces showing I_Na_ in Na_v_1.5-transfected HEK cells at baseline and with flecainide at increasing holding potentials (–100 to –65 mV). **(E)** Reduction in peak I_Na_ with flecainide in Na_v_1.5-transfected HEK cells was enhanced with increased holding potentials. The greatest difference in percent reduction was between –70 and –65 mV. Abbreviations as in Figures 1, 2, and 3.

Atrial *Pitx2c* expression did not modify peak Na^+^ currents (I_Na_) recorded from isolated murine cardiomyocytes at holding potentials ranging from –100 to –65 mV (Figures 5A to 5C). Peak I_Na_ was reduced at more depolarized holding potentials (Figure 5). Flecainide inhibited I_Na_ better at more positive holding potentials (inhibition at –70 mV: 68 ± 5%; inhibition at –65 mV: 75 ± 5%; n = 86 cells from n = 17 atria) in cells from murine atria with normal or reduced *Pitx2c* expression, suggesting that the greater efficiency of flecainide in atria with reduced *Pitx2c* expression is secondary to RMP depolarization (Figure 5C). Consistent with this, flecainide inhibited human Na_v_1.5 channels expressed in HEK cells more potently at more depolarized test potentials (–65 to –75 mV) (Figures 5D and 5E).

**Figure 6:**
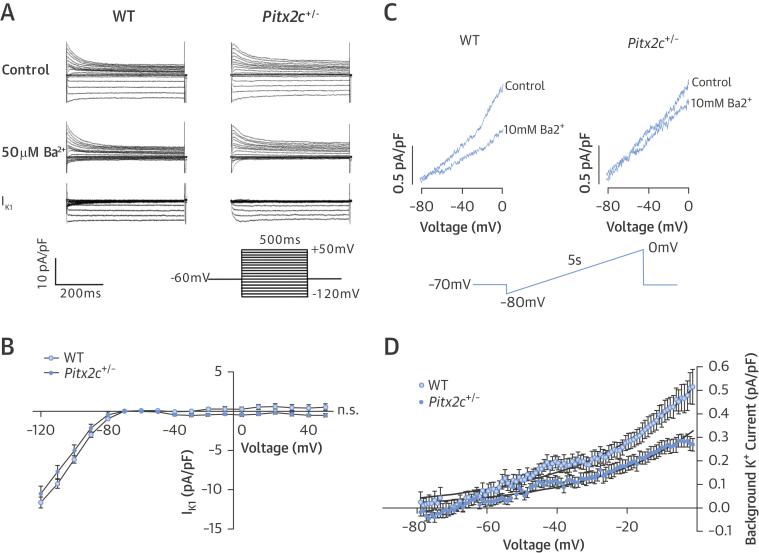
Currents Responsible for RMP **(A)** Representative raw traces showing I_K1_ currents (50 μmol/l Ba^2+^ sensitive) in isolated WT and *Pitx2c*^+/–^ LA cardiomyocytes at test potentials ranging from –120 to 50mV. **(B)** No difference is seen in the I_k1_ current/voltage relationship for LA cardiomyocytes between WT and *Pitx2c*^+/–^ LA at test potentials ranging from –120 to 50 mV. **(C)** Representative raw traces show background current response to 10 mmol/l Ba^2+^ in isolated WT and *Pitx2c*^+/–^ LA cardiomyocytes. **(D)***Pitx2c*^+/–^ cardiomyocytes had significantly reduced background K^+^ (10 mmol/l Ba^2+^ sensitive) currents than WT cardiomyocytes. *p < 0.05. Abbreviations as in Figures 1, 2, and 3.

Background K^+^ currents, which include TASK currents, were reduced in *Pitx2c*^+/–^ murine atria, whereas I_K1_ did not differ between genotypes (Figure 6).

Reduced *Pitx2c* expression did not alter atrial conduction velocities or activation patterns (Online Figures 1A to 1C, Table 6), consistent with published data (8). We found that 1 μmol/l flecainide decreased atrial conduction velocities without differences between wild-type and *Pitx2c*^+/–^ mice (Online Figures 1B and 1C). Calcium transient relaxation times at 50% relaxation were not different between wild-type and *Pitx2c*^+/–^ (Online Figures 1D and 1E). Flecainide 1 μmol/l shortened 50% Ca^2+^ relaxation times by approximately 10% and decreased Ca^2+^ transient amplitude by approximately 50% in murine atria with normal and reduced *Pitx2c* expression (Online Figures 1E and 1F). Additionally, expression of the Na/Ca exchanger Serca2a and Na/K ATPase alpha-1 and alpha-2 subunit protein did not differ between wild-type and *Pitx2c*^+/–^ atria (Online Figure 2).

## Discussion

This study demonstrated that LA *PITX2* mRNA concentrations vary in patients with AF requiring rhythm control therapy (Central Illustration). Furthermore, flecainide increases PRR and suppresses arrhythmias more effectively in atria with halved *Pitx2c* expression, mediated by a more depolarized RMP (Central Illustration). Drug-induced PRR is thought to prevent arrhythmias, as reactivation can then occur only after full recovery of excitability, avoiding slow propagation during the vulnerable period 16, 24, 25. We found similar effects in cells expressing human Na channels and in the Courtemanche–Ramirez–Nattel model of human atrial action potentials.

Thus, this study highlighted modulation of the atrial RMP by *PITX2*, possibly mediated by background currents such as TASK-2, as a target for AAD therapy, including atrial-selective therapy. Furthermore, the results suggested that markers for atrial *PITX2* expression may identify AF patients who benefit from Na-channel blocker therapy (Central Illustration).

Low atrial *PITX2* expression was identified as an important determinant of the antiarrhythmic effects of Na channel blockers. Low LA *Pitx2c* mRNA depolarized atrial RMP (Figure 2), consistent with a previous report (11). A depolarized RMP increased flecainide-induced PRR (Figure 1) 26, 27, 28, 29, 30. The conduction-slowing effect of flecainide was not modulated by reduced atrial *Pitx2c* (Online Figure 1), an important surrogate for drug safety. Both the modeling experiments (Figure 3) and the experiments in HEK cells expressing human Na channels (Figure 5) confirmed that small changes in RMP can markedly modulate Na-channel inhibition.

### Resting membrane potential

Open-state Na-channel blockers such as flecainide and propafenone bind preferentially to Na channels integrated in membranes with slightly depolarized resting potentials, where more channels are in the open or inactivated state 31, 32. Our data can be interpreted as suggesting that AAD combinations that include a Na-channel blocker with a membrane potential modifying substance, such as amiodarone 16, 33 or the combination of dronedarone and ranolazine 29, 34, 35, may have synergistic antiarrhythmic effects because they modulate atrial RMP and thereby enhance the effect of Na-channel blockade. Further studies of such drug combinations and the relationship between their effectiveness and the patient’s atrial *PITX2* mRNA levels are warranted. Our data also suggested that such combined effects may be of special relevance in patients who have a depolarized RMP, such as secondary to low LA *PITX2*. Because *PITX2* expression is confined to the LA in the heart, AAD therapy that leverages modifications in RMP may achieve “atrial-specific” AAD therapy.

RMP is maintained by an intricate balance of different transmembrane currents and is closely related to the potassium equilibrium potential. We identified that *PITX2* modifies expression of the genes encoding K_v_1.6 and TASK-2 (Figure 4). Complete deletion of *PITX2* regulates other potassium and Na channels such as *Kcnj2* 8, 36, which alter the RMP, but these were not responsible for the depolarized RMP observed in our study. Two-pore domain potassium channels, such as TASK-2, contribute to RMP in various cells, including skeletal and cardiac muscle 37, 38. To date, an altered function of the TASK-1 channel and of I_K1_ has been implicated in atrial remodeling and AF 39, 40. This study demonstrated that TASK-2 is expressed in atrial myocardium (Figure 4B), suggesting that a reduced function of TASK-2 could depolarize RMP (Figures 1 and 5) 8, 11, analogous to the effect of TASK-2 in neuronal and cartilage tissue 41, 42.

### Developing clinical markers for patients with depolarized RMP

It will be challenging to directly assess LA RMP in AF patients, but our data suggested that differences in atrial RMP could explain the effectiveness of Na-channel blockers in carriers of common gene variants on chromosome 4q25 (43), although LA *PITX2* levels are modulated by factors other than SNP status (Table 2) (44). It seems desirable to develop and validate drivers that modify RMP and clinical markers for patients prone to a depolarized atrial RMP to select appropriate AADs for individual patients in the future, thus enabling personalized AAD selection 6, 45.

### Study limitations

This study provided robust evidence that LA *PITX2* expression varies in AF patients and that reduced *PITX2c* expression enhances the antiarrhythmic effects of Na-channel blockers by modulating atrial RMP. The study was partly motivated by the assumption that gene variants on chromosome 4q25 modify *PITX2* expression, an assumption that has not been definitively proven 9, 11, 44, 46. Our analysis (Table 2) and that of others indicate that SNP status does not always correlate with *PITX2* levels 47, 48. Our findings are relevant to AAD therapy even if the presumed link between *PITX2* expression and genetic variants on chromosome 4q25 proves elusive. The mechanisms by which reduced *PITX2* mRNA concentrations shorten the LA action potential at high heart rates remain to be fully elucidated 8, 20. Validating our findings in patients is desirable but will be challenging because access to fresh LA cardiomyocytes and LA tissue is limited.

Due to the novelty of our findings, we could not perform a priori power calculations for our mechanistic experiments, and we analyzed several functional parameters to identify potential mechanisms conveying the antiarrhythmic effects of flecainide in atria with low *Pitx2c* concentrations. Our findings thus require independent validation.

## Conclusions

This study shows that low LA *PITX2* mRNA levels increase atrial RMP and thereby increase the effectiveness of flecainide (Central Illustration). This finding calls for appropriately designed clinical studies to assess whether AF patients with low atrial *PITX2* levels respond favorably to Na-channel blockade. Further studies exploring the relevance of TASK channels to atrial RMP also are warranted.

Perspectives **COMPETENCY IN MEDICAL KNOWLEDGE:** *PITX2*, a transcription factor linked to left–right asymmetry in the chest during development, modulates the expression of LA ion channels maintaining the RMP and modulates the antiarrhythmic effects of Na-channel blockers.

**TRANSLATIONAL OUTLOOK:** Clinical studies are needed to assess whether reduced *PITX2* expression identifies patients with AF who respond favorably to Na-channel blocking drugs.

## Figures and Tables

**Central Illustration:**
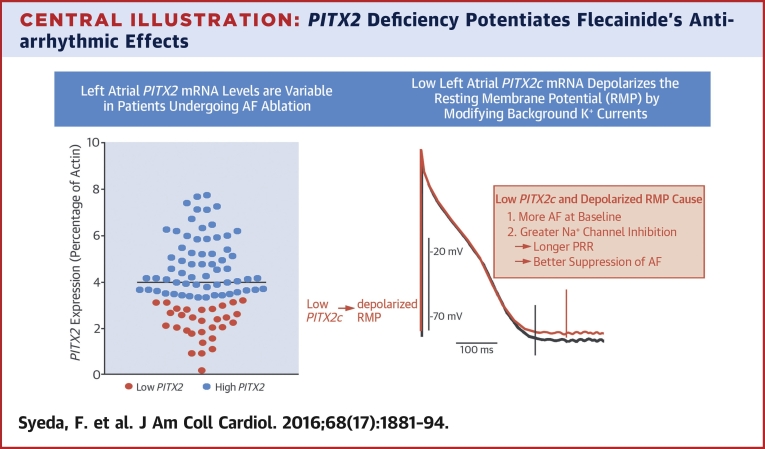
*PITX2* Deficiency Potentiates Flecainide's Antiarrhythmic Effects Paired like homeodomain 2 (*PITX2*) might play an important role in regulating gene expression and electrical function of the left atrium. **(Left)***Pitx2* messenger ribonucleic acid (mRNA) levels are variable in left atrial appendages of patients undergoing thoracoscopic atrial fibrillation (AF) ablation therapy. The differentiation between “low” **(orange)** and “high” **(blue)***PITX2* levels is somewhat arbitrary. **(Right)** A low left atrial *PITX2c* mRNA expression slightly depolarizes left atrial resting membrane potential (RMP). A depolarized RMP, in turn, enhances the antiarrhythmic effect of sodium-channel blockers such as flecainide. PRR = post-repolarization refractoriness.

**Table 1:** Baseline Characteristics (N= 101)∗

Age, yrs	59.7 ± 8.4 (40–76)
Male	79
Congestive heart failure	6
Hypertension	34
Age ≥75 yrs	1
Diabetes	9
Stroke/transient ischemic attack/embolus	10
Vascular disease	10
Female	22
Age ≥65 yrs	31
CHA_2_DS_2_-VASc score	
0	60
1	24
≥2	17
Previous catheter ablation for AF	20
Type of AF	
Paroxysmal	44
Persistent	56
Longstanding persistent	1
AF duration, yrs	6.0 (1–35)
Antiarrhythmic drugs and rate control agents	
Quinidine or disopyramide	4
Flecainide or propafenone	33
Amiodarone, dronedarone, or sotalol	41
Beta blockers	53
Verapamil or diltiazem	17
Digoxin	15
Anticoagulant agents (before PVI procedure)	
Vitamin K antagonists	89
Antiplatelets	6

Values are mean ± SD (range), n, or mean (range).

PVI = pulmonary vein isolation.

∗ Left atrial appendages were collected from these patients with atrial fibrillation (AF).

**Table 2:** *PITX2* mRNA Expression in Left Atrial Appendages From AF Ablation Patients∗

Risk Alleles	25% IQR	Median	75% IQR	Mean	SEM	No. of Patients
0	3.22	3.69	5.22	4.04	0.6	3
1	2.96	4.25	6.25	4.54	0.5	13
2	2.65	3.78	4.75	3.94	0.3	22
3	2.74	3.72	4.92	3.83	0.4	17
4	3.00	4.29	5.41	4.39	0.5	10
5	1.96	2.66	4.66	3.10	0.7	4
6	4.95	4.95	4.95	4.95	0.0	1

IQR = interquartile range; LA = left atrium; other abbreviations as in Table 1.

∗ This dataset was grouped according to the number of risk single nucleotide polymorphism (SNP) alleles for AF on chromosome 4q25 (rs2200733, SNP2 rs6838973, rs1448818 [13]). Although *PITX2* mRNA is numerically lower in patients with 5 or 6 risk alleles, we did not find a *PITX2* mRNA gradient according to AF risk.

**Table 3:** Effect of Flecainide on Refractoriness and Repolarization in Mouse Hearts

Paced CL, ms	Wild-Type						*Pitx2c*^+/–^					
	120		100		80		120		100		80	
	Baseline	Flecainide	Baseline	Flecainide	Baseline	Flecainide	Baseline	Flecainide	Baseline	Flecainide	Baseline	Flecainide
LA ERP, ms												
	23.5 ± 2.3 (11)	29.8 ± 3.0 (11)	22.2 ± 2.1 (11)	29.6 ± 3.3 (11)	21.9 ± 2.4 (10)	28.7 ± 3.5∗ (10)	30.5 ± 2.4 (11)	38.5 ± 3.3∗ (11)	28.0 ± 2.3 (13)	40.2 ± 2.8∗ (13)	27.5 ± 2.5 (13)	41.2 ± 3.0∗† (13)
LA monophasic APD, ms												
APD_50_	10.2 ± 1.3 (8)	14.5 ± 1.7 (8)	10.8 ± 1 (8)	11.9 ± 1.6 (8)	10.4 ± 0.7 (7)	12.0 ± 1.1 (7)	12.4 ± 1.1 (15)	14.4 ± 1.3 (15)	11.5 ± 1.0 (15)	12.4 ± 1.1 (15)	10.6 ± 0.9 (11)	10.3 ± 1.0 (11)
APD_70_	17.8 ± 2.2 (9)	23 ± 2.1 (9)	18.4 ± 1.6 (9)	18.7 ± 2.2 (9)	18.1 ± 1.2 (8)	18.1 ± 1.9 (8)	18.0 ± 1.6 (15)	19.2 ± 1.8 (15)	16.0 ± 1.4 (13)	16.2 ± 1.4 (13)	14.9 ± 1.0† (10)	13.1 ± 0.7 (10)
APD_90_	31.3 ± 3.0 (8)	37.4 ± 2.8 (8)	31.5 ± 2.5 (9)	29.9 ± 2.9 (9)	31.0 ± 1.4 (8)	28.4 ± 2.7 (8)	28.3 ± 2.2 (13)	29.9 ± 2.2 (13)	27.4 ± 2.2 (13)	26.6 ± 1.5 (13)	26.8 ± 1.7 (10)	23.1 ± 1.4 (10)
LA transmembrane APD, ms												
APD_30_	4.5 ± 0.1 (30)	5.5 ± 0.3 (22)	4.5 ± 0.1 (30)	5.4 ± 0.3 (22)	4.4 ± 0.1 (30)	5.2 ± 0.3 (22)	4.0 ± 0.1 (31)	4.7 ± 0.2 (24)	3.9 ± 0.1 (31)	4.6 ± 0.2 (24)	3.8 ± 0.1† (31)	4.4 ± 0.2 (24)
APD_50_	6.7 ± 0.2 (30)	8.2 ± 0.5 (22)	6.6 ± 0.2 (30)	8.0 ± 0.4 (22)	6.4 ± 0.2 (30)	7.8 ± 0.5 (22)	5.9 ± 0.2 (31)	7.1 ± 0.4 (24)	5.7 ± 0.2 (31)	7.0 ± 0.4 (24)	5.6 ± 0.2† (31)	6.7 ± 0.3 (24)
APD_70_	10.5 ± 0.4 (30)	12.7 ± 0.8 (22)	10.1 ± 0.4 (30)	12.1 ± 0.7 (22)	9.6 ± 0.4 (30)	11.8 ± 0.7 (22)	8.9 ± 0.4 (31)	10.7 ± 0.6 (24)	8.6 ± 0.4 (31)	10.3 ± 0.6 (24)	8.3 ± 0.3† (31)	9.8 ± 0.5 (24)
APD_90_	20.9 ± 1.0 (30)	23.4 ± 1.5 (22)	19.9 ± 0.9 (30)	22.2 ± 1.4 (22)	18.4 ± 0.8 (30)	21.6 ± 1.3 (22)	17.6 ± 0.9 (31)	20.3 ± 1.1 (24)	16.5 ± 0.8 (31)	19.2 ± 1.0 (24)	15.7 ± 0.8† (31)	17.9 ± 0.9 (24)
LA optical APD, ms												
APD_30_	6.1 ± 0.3 (10)	7.3 ± 0.6 (6)	6.4 ± 0.8 (10)	5.9 ± 1.0 (6)	6.1 ± 0.4 (10)	6.9 ± 1.3 (6)	4.9 ± 0.4 (10)	7.7 ± 0.9 (8)	4.6 ± 0.3 (10)	5.4 ± 0.7 (8)	4.3 ± 0.4† (10)	5.7 ± 0.7 (8)
APD_50_	8.5 ± 0.6 (10)	10.7 ± 1.2 (6)	8.9 ± 1.1 (10)	8.5 ± 1.2 (6)	8.3 ± 0.7 (10)	10.3 ± 1.8 (6)	6.9 ± 0.4 (10)	10.0 ± 1.0 (8)	6.6 ± 0.4 (10)	8.1 ± 0.9 (8)	6.1 ± 0.4† (10)	8.0 ± 0.9 (8)
APD_70_	11.7 ± 1.2 (10)	15.0 ± 2.1 (6)	12.5 ± 1.5 (10)	12.8 ± 1.7 (6)	11.5 ± 1.1 (10)	14.4 ± 2.5 (6)	9.4 ± 0.0 (10)	13.3 ± 1.2 (8)	9.4 ± 0.6 (10)	11.6 ± 1.5 (8)	9.1 ± 0.5† (10)	11.2 ± 1.2 (8)

Values are mean ± SEM (number of atria).

APD = action potential duration; CL = cycle length; ERP = effective refractory period; *Pitx2c*^+/–^ = *PITX2* deficient; other abbreviations as in Tables 1 and 2.

∗ p < 0.05 vs. baseline.

† p < 0.05 vs. wild-type.

**Table 4:** Electrophysiological Effects of Sotalol

**Paced CL, ms**	Wild-Type				*Pitx2c*^+/–^			
	**120**		**100**		**120**		**100**	
	**Baseline**	**Sotalol**	**Baseline**	**Sotalol**	**Baseline**	**Sotalol**	**Baseline**	**Sotalol**
LA ERP, ms								
	38.7 ± 7.8 (7)	33.9 ± 6.3 (7)	32.2 ± 6.1 (6)	29.2 ± 5.3 (6)	39.3 ± 4.0 (4)	26.8 ± 3.5 (4)	37.0 ± 5.7 (4)	24.0 ± 3.7 (4)
LA APD, ms								
APD_50_	11.5 ± 1.2 (9)	13.4 ± 1.2 (9)	10.9 ± 2.0 (7)	12.2 ± 1.3 (7)	10.8 ± 1.1 (7)	11.2 ± 1.0 (7)	8.3 ± 0.9 (4)	11.1 ± 1.7 (4)
APD_70_	17.6 ± 2.2 (9)	20.0 ± 1.9 (9)	16.0 ± 1.3 (7)	18.2 ± 2.3 (7)	16.5 ± 1.6 (7)	17.3 ± 1.2 (7)	13.0 ± 1.5 (4)	17.0 ± 1.9 (4)
APD_90_	30.7 ± 3.2 (9)	33.5 ± 2.7 (9)	29.0 ± 1.9 (7)	30.9 ± 3.2 (7)	29.7 ± 2.7 (7)	31.2 ± 2.0 (7)	23.8 ± 2.8 (4)	29.6 ± 2.9 (4)

Values are mean ± SEM (number of atria).

Abbreviations as in Tables 2 and 3.

**Table 5:** Electrophysiological Effects of Reduced Sodium Conductance in a Human Atrial Model

Paced CL, ms	Wild-Type Model		*Pitx2c* Deficiency Model	
	500	1,000	500	1,000
RMP, mV				
g_Na_, %				
100	-79.92	-81.28	-77.90	-79.61
50	-79.60	-81.12	-77.33	-79.37
40	-79.43	-81.01	-76.99	-79.23
APD at repolarization to -60 mV, ms				
g_Na_, %				
100	217	253	206	226
50	239	266	233	239
40	248	273	245	245
ERP, ms				
g_Na_, %				
100	266	301	261	280
50	308	335	316	320
40	327	352	342	339
PRR, ms				
g_Na_, %				
100	49	48	55	54
50	69	69	83	81
40	79	79	97	94
Conduction velocity, cm/s				
g_Na_, %				
100	49.5	50.0	50.3	50.5
50	36.9	37.0	37.5	37.5
40	32.9	32.7	33.0	33.1

g_Na_ = reduced sodium conductance; PRR = post-repolarization refractoriness; RMP = resting membrane potential; other abbreviations as in Table 3.

**Table 6:** Electrical Activation Time and Conduction Velocity in Isolated Atria in the Presence of Flecainide (1 μmol/l)

	Wild-Type					*Pitx2c*^+/–^				
Paced CL, ms	1,000	300	120	100	80	1,000	300	120	100	80
Activation time (isolated left atrium), ms	6 ± 0.3 (22)	6 ± 0.3 (22)	9 ± 0.5 (22)	12 ± 1.0 (22)	16 ± 1.4 (22)	6 ± 0.2 (24)	7 ± 0.3 (24)	12 ± 0.9 (24)	13 ± 0.9 (24)	18 ± 1.2 (24)
Conduction velocity (optical mapping), cm/s	—	30 ± 1.8 (8)	25 ± 2.4 (8)	25 ± 1.9 (8)	23 ± 1.9 (8)	—	29 ± 1.5 (8)	26 ± 1.6 (8)	25 ± 1.6 (8)	23 ± 1.6 (8)

Values are mean ± SEM (number of atria).

— = not applicable; other abbreviations as in Table 3.
