# Supplementary material for: PITX2 Modulates Atrial Membrane Potential and the Antiarrhythmic Effects of Sodium-Channel Blockers
Source: J Am Coll Cardiol. 2016 Oct 25;68(17):1881–94. doi: 10.1016/j.jacc.2016.07.766 (PMC5075046; doi:10.1016/j.jacc.2016.07.766)
Supplement: Online Data [file mmc1.pdf]

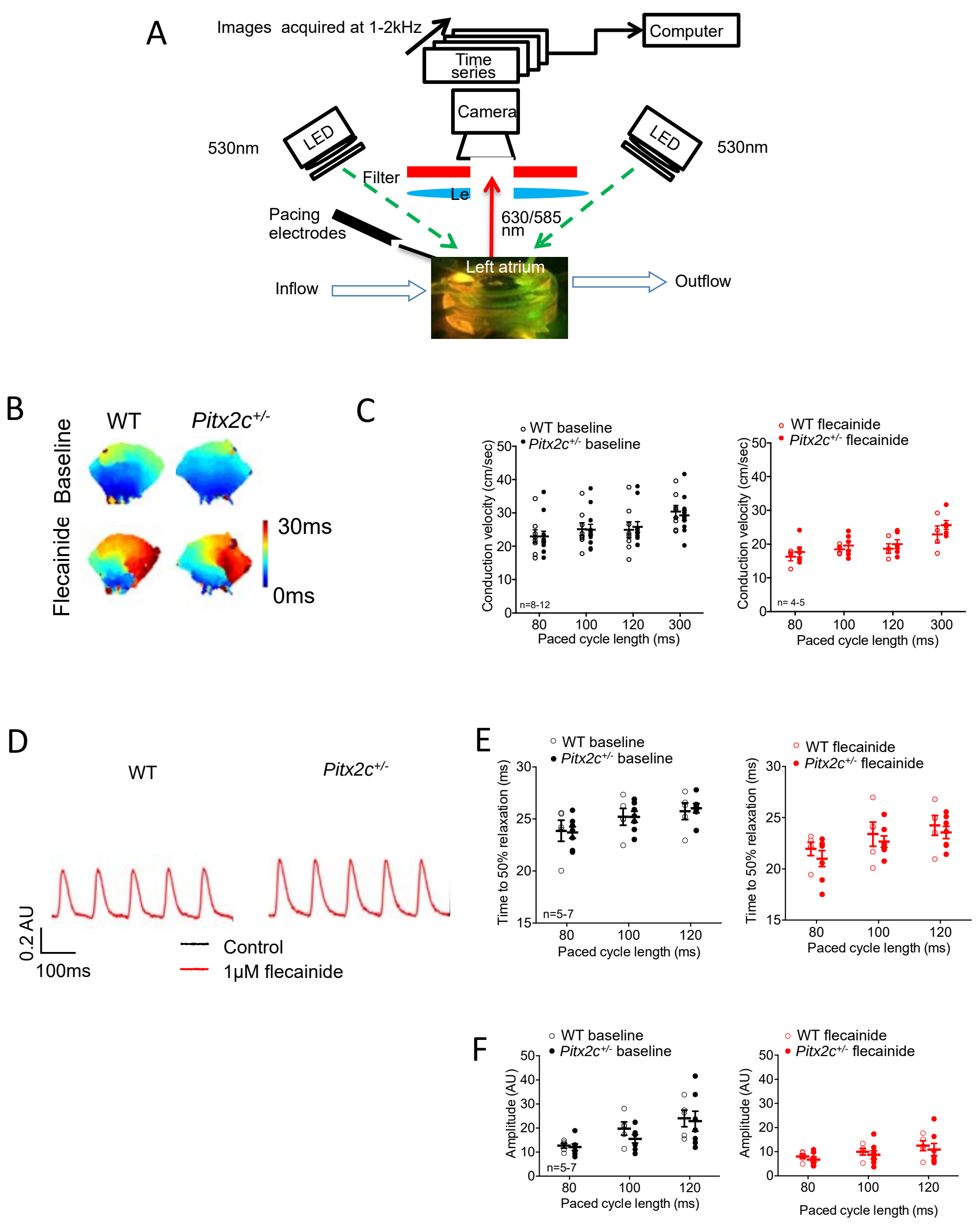


Supplementary Figure 1

## Supplementary Figure 1

**A** Experimental setup of the high density optical activation mapping system. This system was also used to measure $Ca^{2+}$ transients.

**B** Representative electrical activation maps of wildtype (WT) and *Pitx2c*$^{+/-}$ LA before and after flecainide, recorded at 100ms cycle length pacing. Flecainide causes conduction slowing in both genotypes to the same degree.

**C** Conduction velocity determined by optical activation mapping in superfused *Pitx2c*$^{+/-}$ and wildtype LA, measured at 80-300ms paced CL. Baseline (black): wildtype n=5; *Pitx2c*$^{+/-}$ n=11. Flecainide (red): wildtype: n=4; *Pitx2c*$^{+/-}$ n= 8. Individual values are displayed. Data points for each genotype have been offset at each cycle length for clarity.

**D** Representative calcium transients recorded during 100ms cycle length pacing at baseline (black) and with 1µmol/L flecainide (red) from isolated superfused wildtype and *Pitx2c*$^{+/-}$ LA . Calcium transients did not differ between genotypes with or without flecainide. Horizontal line= 40 ms.

**E** Time to 50% calcium relaxation at baseline and after 1µmol/L flecainide in LA. Calcium relaxation time did not differ between genotypes with or without flecainide. Baseline (black): wildtype n=5; *Pitx2c*$^{+/-}$ n=7. Flecainide (red): wildtype: n=5; *Pitx2c*$^{+/-}$ n= 7.

**F** Calcium signal amplitude at baseline and after 1 µmol/L flecainide in LA. AU= arbitrary units; fold-change in fluorescence. Baseline (black): wildtype n=5; *Pitx2c*$^{+/-}$ n=7. Flecainide (red): wildtype: n=5; *Pitx2c*$^{+/-}$ n= 7. Data points for each genotype have been offset at each cycle length for clarity.

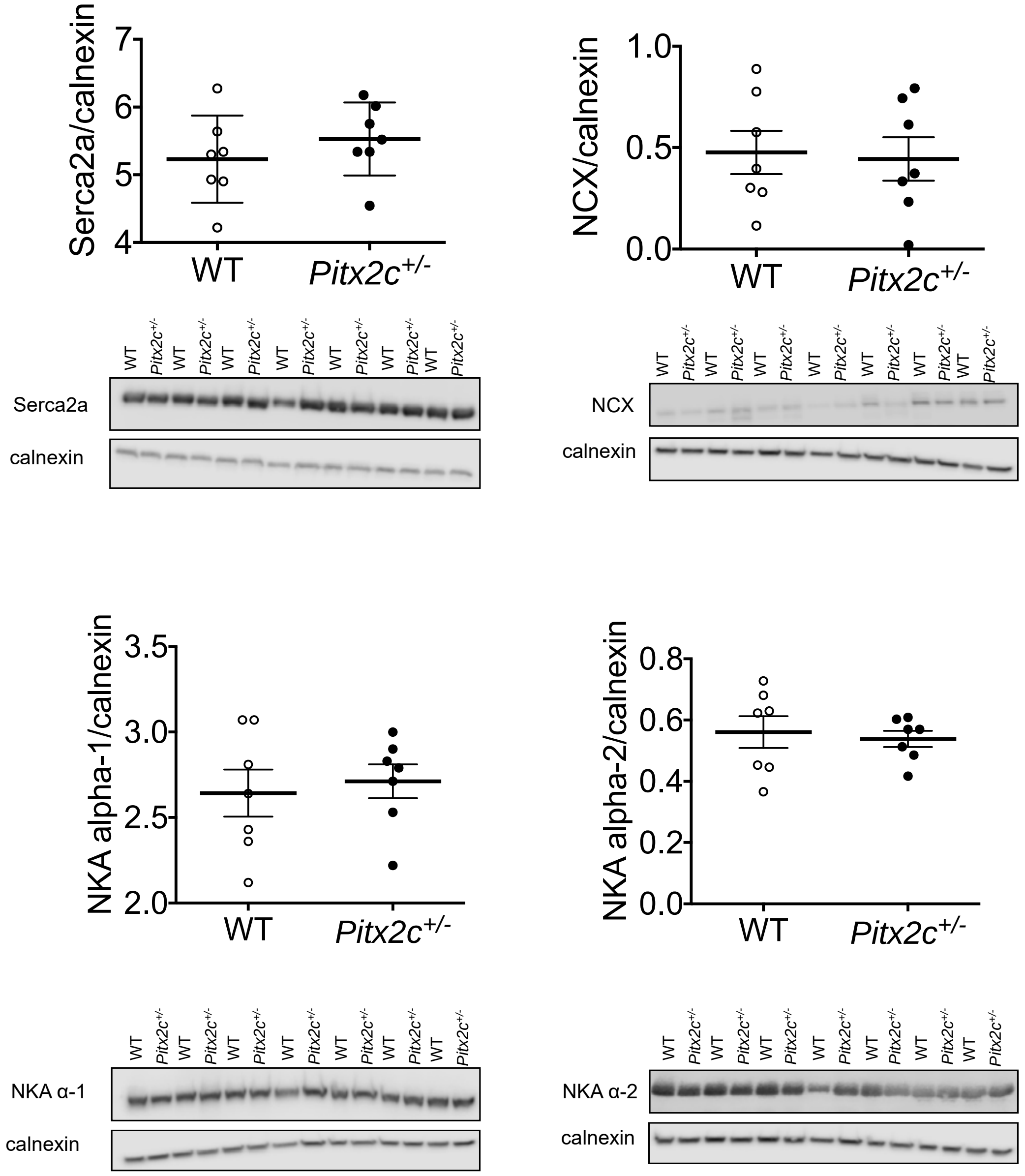


Supplementary Figure 2

**Supplementary Figure 2**

Protein concentrations of Na/Ca exchanger, Serca2a, Na/K ATPase alpha-1 and alpha-2 proteins, relative to calnexin (arbitrary units) in left atrial homogenates of wild type (WT) and heterozygous *Pitx2c*$^{+/-}$ mice. Representative immunoblots are displayed below the corresponding dot plot. WT = 7, *Pitx2c*$^{+/-}$ = 7.

**Supplementary Table:** Quantification of mRNA of ion channels in left atria from *Pitx2c*$^{+/-}$ and wild-type mice. Expression levels were measured relative to wildtype sample 1. * and boldface indicate p<0.05

| **Gene** | **Protein** | **WT Rq$_{control}$ ±SEM** | ***Pitx2c*$^{+/-}$ Rq$_{control}$ ±SEM** | **Mean CT ±SEM** |
|---|---|---|---|---|
| *Pitx2* | PITX2 | 1 ± 0.13 | 0.77 ± 0.09 | 26.4* ± 0.12 |
| *Kcna6* | $K_v$1.6 | 1 ± 0.15 | 0.65 ± 0.09 | 27.2* ± 0.17 |
| *Kcnk5* | TASK-2 | 1 ± 0.1 | 0.71 ± 0.12 | 28.6* ± 0.13 |
| *Actb* | ACTB | 1 ± 0.19 | 0.84 ± 0.09 | 19.7 ± 0.06 |
| *Cacna1c* | Cav1.2 | 1 ± 0.08 | 1.03 ± 0.08 | 22.7 ± 0.11 |
| *Cacna2d2* | CACNA2D | 1 ± 0.11 | 1.27 ± 0.15 | 25.1 ± 0.17 |
| *Casq2* | CSQ2 | 1 ± 0.06 | 0.98 ± 0.06 | 19.9 ± 0.18 |
| *Cul7* | P185 | 1 ± 0.07 | 0.96 ± 0.08 | 25.5 ± 0.1 |
| *Itpr2* | AI649341 | 1 ± 0.14 | 0.87 ± 0.07 | 25.3 ± 0.11 |
| *Kcna3* | $K_v$1.3 | 1 ± 0.8 | 0.16 ± 0.03 | 31.6± 0.17 |
| *Kcna4* | $K_v$1.4 | 1 ± 0.12 | 0.94 ± 0.08 | 27.7 ± 0.16 |
| *Kcnc4* | $K_v$3.4 | 1 ± 0.43 | 0.85 ± 0.39 | 32.3 ± 0.43 |
| *Kcnj2* | IRK1 | 1 ± 0.07 | 0.91 ± 0.07 | 23.9 ± 0.12 |
| *Kcnj3* | GIRK-1 | 1 ± 0.05 | 0.98 ± 0.03 | 19.5 ± 0.13 |
| *Kcnj5* | GIRK4 | 1 ± 0.08 | 0.95 ± 0.07 | 21.5 ± 0.11 |
| *Ryr2* | RYR2 | 1 ± 0.09 | 0.88 ± 0.06 | 18.4 ± 0.11 |
| *Scn1b* | SCN1B | 1 ± 0.08 | 0.88 ± 0.06 | 23.6± 0.1 |
| *Scn4a* | $Na_v$1.4 | 1 ± 0.1 | 1.25 ± 0.12 | 24.4 ± 0.11 |
| *Scn5a* | $Na_v$1.5 | 1 ± 0.07 | 0.98 ± 0.1 | 21.2± 0.1 |
| *Scn7a* | $Na_v$2 | 1 ± 0.13 | 0.8 ± 0.04 | 24 ± 0.09 |
| *Trpc1* | TRP1 | 1 ± 0.07 | 0.94 ± 0.06 | 26.5± 0.1 |
| *Trpc6* | TRP-6 | 1 ± 0.13 | 0.88 ± 0.14 | 31.1 ± 0.15 |
| *Trpm7* | TRPM7 | 1 ± 0.12 | 0.84 ± 0.04 | 23.7 ± 0.12 |

**Supplementary methods**

**Electrophysiological study in the isolated heart.**

Mice were terminally anaesthetized with 400mg/kg pentobarbital sodium and heparinized. Their hearts were rapidly excised, mounted on a Langendorff apparatus (Hugo Sachs, Germany) and retrogradely perfused with 37°C Krebs-Henseleit (KH) containing (in mmol/l): NaCl 118; $NaHCO_3$ 24.88; $KH_2PO_4$ 1.18; Glucose 5.55; Na-Pyruvate 5; $MgSO_4$ 0.83; $CaCl_2$ 1.8; KCl 3.52 (95% $O_2$–5% $CO_2$, pH 7.4) at constant perfusion pressure (100 ± 5 mmHg) and coronary flow (4 ± 0.5ml/min).

A 2.0 French octapolar mouse electrophysiological catheter (0.5 mm electrode spacing, CIB'ER MOUSE, NuMED, USA) was inserted into the right atrium and right ventricle for pacing and recording intracardiac electrograms. Left atrial epicardial monophasic action potentials (MAP) were recorded using custom-made electrodes, preamplified using a DC-coupled pre amplifier (Model 2000, EP Technologies, USA), recorded at 2 kHz sampling frequency, and analyzed offline (EMKA technologies, France). Inducibility of atrial arrhythmias (triplets, salvos and AF) and effective refractory periods, with and without flecainide acetate (Sigma, 1µmol/L) and sotalol hydrochloride (Sigma, 10µmol/L), were determined using 120 to 80 ms paced cycle lengths and premature single RA extrastimulus. Activation times and action potential durations at different repolarization levels were analyzed.

**Transmembrane action potential recordings in isolated, superfused left atria.**

Transmembrane action potentials were recorded using borosilicate glass microelectrodes, filled with 3 M KCl (tip resistance 15–30 MΩ), from intact

superfused left atria isolated from mice under inhalation anaesthesia (2.5-4% isoflurane in $O_2$, 1.5L/min). Voltage signals were amplified (Axoclamp 2B; Molecular Devices, USA), digitized, displayed and analyzed using spike2 software (Cambridge Electronic Design, UK) at 20 kHz sampling frequency. LA were paced at 2× diastolic threshold through bipolar platinum electrodes. After 15 min pacing for equilibration, preparations were paced successively at 1000ms to 80ms. Action potentials were analyzed following 200 beats at each frequency to ensure steady state prior to recording.

**$Na^+$ current recordings in murine left atrial cardiomyocytes and HEK293 cells expressing the human $Na_v1.5$ channel**

Murine hearts were removed under terminal anaesthesia and cell isolation was performed as published. For $I_{Na}$ recordings, murine left atrial cardiomyocytes or HEK293 cells stably expressing the human $Na_v1.5$ channel (SB Ion Channels, Glasgow, UK) were superfused at 5ml.min$^{-1}$, 22±0.5 °C with a solution containing in mM: NaCl 130, CsCl 5, HEPES 10, $CaCl_2$ 1.8, $MgCl_2$ 1.2 and glucose 10, pH 7.4 (CsOH). 100µM $CdCl_2$ was added to block L-type $Ca^{2+}$ currents. Whole cell patch clamp recordings were obtained in voltage clamp mode using borosilicate glass pipettes (tip resistance 1–3 MΩ, pipette solution CsCl 115, NaCl 5, HEPES 10, EGTA 10, MgATP 5, $MgCl_2$ 0.5 and TEA 10, pH 7.2). $Na^+$ currents were elicited at 100ms steps to -10mV from holding potentials of -100 to -65mV using an Axopatch 1D amplifier (Molecular Devices, USA) and a CED micro1401 driven by Signal v6 (CED, UK).

Standard $I_{K1}$ currents were isolated using 50µM $BaCl_2$ and applying 10mV step depolarisations (500ms) from -120mV to +50mV. For background $K^+$ current measurements, the external solution contained in mM: NaCl 130, KCl 5.4, $CaCl_2$ 1, $MgCl_2$ 1, HEPES 10, TEA 10, 4-AP 5, glibenclamide (2µM), $BaCl_2$ (100µM), $NiCl_2$ 2 and glucose 5.5 (pH 7.4 with NaOH), to block voltage-gated $K^+$ and $Ca^{2+}$ channels and $I_{K1}$ and $I_{KATP}$. Currents were measured in response to a 5s voltage ramp from -80 to 0mV. Background TASK-like $K^+$ current was isolated with 10mM $BaCl_2$.

**Quantitative immunoblotting of atrial tissue**. Frozen LA were homogenized in 30 ml of homogenization buffer per gram of tissue (100 mM Tris, pH 7.4, 2 mM sodium vanadate, 5 mM sodium fluoride, 1× protease inhibitor cocktail tablet/50 mL, 4°C). Samples were separated by electrophoresis on a 10% or 15% SDS polyacrylamide gel. Proteins were transferred to PVDF membranes (0.45µm; GE Healthcare, UK) using semidry blotting (Trans-Blot® Turbo™ Transfer System BioRad). Immunoblots were blocked with 5% non-fat milk in PBS-Tween overnight at 4°C. Blots were incubated for 1-1.5 hours at room temperature with primary antibodies raised against: TASK-2 (1:200; Alomone), $K_v1.6$ (1:200; Alomone), Na/K ATPase alpha-1 (1:10000; Milipore, MA, USA), Na/K ATPase alpha-2 (1:5000; Milipore, MA, USA), Na/Ca exchanger 1 (1:1000; Swant, Switzerland), Serca2a (1:5000; Badrilla, UK) and calnexin (1:2000, ab22595 Abcam). Blots were incubated with $Na_v1.5$ antibody (1:200, Alomone) overnight at 4°C. After incubation with HRP-labeled secondary antibodies, blots were developed using enhanced chemiluminescence (Amersham Pharmacia Biotech) on

Odyssey Fc imager (Li-Cor). Calnexin was used as a loading control. Signals from *Pitx2c*$^{+/-}$ LA samples were normalized to wildtype samples signals on the same gels.
